# Self-Esteem Group: Useful Intervention for Inpatients with Anorexia Nervosa?

**DOI:** 10.3390/brainsci9010012

**Published:** 2019-01-13

**Authors:** James Adamson, Cansu Ozenc, Claire Baillie, Kate Tchanturia

**Affiliations:** 1 Department of Psychological Medicine, Institute of Psychiatry, Psychology and Neuroscience, King’s College London, London SE5 8AF, UK; james.adamson@kcl.ac.uk; 2 South London and Maudsley NHS Foundation Trust, London BR3 3BX, UK; c.ozenc@nhs.net (C.O.); Claire.Baillie@slam.nhs.uk (C.B.); 3 Department of Psychology, Illia State University, Tbilisi 0162, Georgia

**Keywords:** self-esteem, anorexia nervosa, cognitive behavioral therapy, group therapy, inpatient treatment, eating disorder

## Abstract

Low self-esteem is a common feature in Anorexia Nervosa (AN) and has been hypothesised to act as a predisposing, precipitating, and perpetuating factor. The aim of this study was to assess the effectiveness of a Cognitive Behavioural Therapy (CBT)-based self-esteem group in a naturalistic setting of patients with AN in an inpatient treatment programme. Included in this study were 119 female patients diagnosed with AN, with all participants completing self-report questionnaires before and after the intervention. The group consisted of five to six weekly sessions. The self-esteem group led to a statistically significant improvement in self-esteem, which could not be explained by an increase in BMI alone, suggesting that the group is facilitating positive changes within an AN group. The group also had a small effect on improving patients self-perceived ability to change. These findings suggest that the brief self-esteem group has some benefits in improving patients’ self-esteem/self-efficacy and should be replicated in the future with a control condition to confirm findings.

## 1. Introduction

Attempts have been made to explore various group treatment modalities that are effective in improving the symptoms and maintaining factors for patients with AN [1,2]. Although there is no evidence that the treatments offered in a group setting are superior to individual treatments, there is a growing body of literature that recognises group therapy as an effective, meaningful, and cost effective form of psychological intervention [3,4]. It has been recognised that group interventions provide a safe therapeutic setting to learn from others, practice communication skills, and provide psychoeducational and homework materials [5]. Although previous research suggests that positive outcomes were evidenced in the implementation of Interpersonal Therapy, Dialectical Behaviour Therapy, Cognitive Remediation Therapy, body image, perfectionism, and motivational models [6,7,8,9,10,11], much uncertainty exists about the most efficacious intervention for AN if offered in a group format [12].

Several definitions of self-esteem have been proposed; it can be broadly defined as a positive or negative attitude towards oneself. Low self-esteem encompasses a lack of respect for oneself as well as feelings of insufficiency, unworthiness, and failure [13]. Fennell (1998) has proposed a cognitive behavioural model that suggests that one’s early life experiences can shape current perceptions of oneself whilst cognitive maintenance cycles make it difficult for the individual to change their perception [14]. Low self-esteem is a core symptom of AN and has been hypothesised to be a risk factor as well as a predisposing, precipitating, and perpetuating factor [15]. Furthermore, it is considered to play an important role in the development, treatment, and recovery from AN [16,17]. Button (1993) has asserted that low self-esteem is an important personality characteristic in determining whether an individual is at risk of developing an eating disorder [18]. It has been well established that individuals with AN have low self-esteem relative to non-eating-disorder controls [19,20,21,22,23]. A longitudinal study of patients with AN has also found that low self-esteem is linked with lower overall well-being [24]. Fairburn et al. (2003) has argued that self-esteem maintains components of eating disorder psychopathology, particularly contributing to concerns around shape, weight, and dietary restraint [20]. Furthermore, it has been found that low self-esteem is a weak prognostic indicator in the treatment of eating disorders [20,24,25]. Button and Warren (2002) have demonstrated that problems with low self-esteem persist even after weight restoration and positive changes in eating behaviour.

There is uncertainty about the direction of causality in the relationship between self-esteem and eating disorders. Silverstone (1992) has argued that low self-esteem pre-dates the onset of the eating disorder and viewed eating disorders as a symptom of low self-esteem [26]; this view has been supported by further research [27,28]. Newns et al. (2003) have suggested that treatments targeted solely on self-esteem have a considerable potential in reducing eating disordered attitudes [27]. Furthermore, Collin et al. (2016) have explored the associations between multidimensional self-esteem and eating disorder symptoms among adults participating in an inpatient treatment programme for AN [29]. They have found that self-esteem improved over the course of the treatment; however these improvements were domain-specific, with small effect sizes. Relevant literature is presented in Table 1. 

Despite an increasing interest in the role of self-esteem in the treatment of AN, there is still a need to evaluate group formats targeting self-esteem using different protocols to generate more evidence. The aim of this study is to examine the feasibility of a CBT-based self-esteem group in a naturalistic setting of inpatients with AN. We hypothesised that over the course of the CBT-based self-esteem group, participants’ self-reported self-esteem measures would improve.

## 2. Materials and Methods

### 2.1. Participants 

This naturalistic sample, collected over 7 years, consisted of 119 patients aged between 18 to 54 years, with a primary diagnosis of anorexia nervosa, diagnosed by a consultant psychiatrist based on DSM IV and 5 criteria. Participants were recruited from the South London and Maudsley NHS Foundation Trust inpatient treatment programme. All inpatients through the years of 2011–2018 were offered the opportunity to attend the CBT-based self-esteem group. All patients who attended the group consented to the study and those who completed the baseline assessments were included in this study. As the data was collected from a clinical programme, all participants received additional clinical input during their inpatient treatment, including nutritional and medical interventions as well as other therapeutic groups and individual therapies [5]. All participants were female due to it being a single-gender inpatient ward.

All patients attended at least five out of six group sessions to be included in the analysis. Where participants took part in the self-esteem group more than once, the earliest data point was taken. The Study was approved by the hospital clinical governance committee, South London and Maudsley NHS foundation Trust (N-201101) and all participants consented for their data to be used for anonymous publications.

### 2.2. CBT-Based Self-Esteem Group 

The self-esteem group was designed to address different components of low self-esteem, low confidence, low self-worth, and low assertiveness. Cognitive behavioural group therapy aims to address psychological distress and maladaptive behaviour by facilitating changes on individuals’ style of thinking [31]. A number of resources, including Fennell’s self-help book “Overcoming low self-esteem” (2006) and experiential exercises were modified and adapted to facilitate discussions in a group format [32]. 

The intervention consisted of five or six sessions and was delivered once a week by a clinically qualified psychologist and co-facilitated by one of the multidisciplinary team members. In 2018, the group was reduced to five sessions, covering the same material in a reduced period of time. Table 2 provides information on the outline of the group in its current five-session format. The focus of the group sessions was to become aware of and reflect on critical and negative thinking [12]. The following specific strategies were used for the group format: psychoeducation, practical and interactive exercises, time spent for reflection and discussion, and homework tasks to encourage further exploration outside the sessions. The group utilised visual metaphors of a poisoned parrot and proud peacock to help patients continue reflections outside the sessions; these metaphors were further reinforced by having images of these around the unit.

The CBT intervention did not specifically target motivation; however, the cognitive approach, designed to facilitate patients to gain insight into their illnesses, albeit indirectly, aimed at increasing patients’ motivation.

### 2.3. Measures 

BMI (kg/m^2^) is a measure of body weight in relation to height. BMI was taken from patients’ clinical notes on the day of the start and end of the intervention.

The Rosenberg Self-Esteem Scale (RSES) measures one’s feelings of overall self-worth with an equal number of positively (e.g., ‘On the whole, I am satisfied with myself’) and negatively (e.g., ‘I feel I do not have much to be proud of’) worded items. The responses are rated on a four-point Likert scale indicating level of agreement (1–4, ranging from ‘strongly disagree’ to ‘strongly agree’). A total scale score is computed by summing the item responses for the 10 items. Higher scores represent better self-esteem, and scores below 15 suggest low self-esteem. It is the most widely used scale to measure self-esteem in the current literature. In the current study, the Cronbach’s alpha coefficient was 0.84.

The Motivational Ruler (MR) is a two-item visual analogue scale measuring patients self-perceived motivation to change and ability to change with higher scores indicating high levels of motivation.

Autism Spectrum Quotent, short version (AQ-10) is a ten-item likert scale originally designed as a screening questionnaire for full Autism Spectrum Disorder (ASD) assessments. Patients completed the AQ-10 on admission to the programme. A score equal to or above 6 was used to indicate high levels of ASD traits and to group participants into high and low scorers [33].

### 2.4. Statistical Analysis 

Data management and all statistical analyses were performed using R (R Development Core Team, 2008). The data was checked to ensure that the necessary assumptions for utilising parametric statistics were met. Normality checks were carried out and confirmed with histograms. A linear mixed effects analyses was conducted to investigate changes from pretreatment to posttreatment in the RSES Scale and motivational ruler. Time (baseline, end of treatment) was entered as a fixed effect predictor and BMI was included as a covariate to explore its potential impact on self-esteem and motivation. The effect size (ES) estimates were interpreted as small (≥ |0.2|), medium (≥ |0.4|), and large (≥ |0.8|). Secondary analysis was conducted firstly using AQ-10 scores ≥ 6 as a grouping variable to compare improvement in those with low and high levels of ASD traits. Furthermore, age and duration of illness were included as covariates to explore their potential impact on self-esteem and motivation.

## 3. Results

The mean age of patient group was 26 (range 18 to 54) with mean age of illness onset of 17 years (SD = 5.83) and a mean illness duration of 9 years (SD = 7.92). Due to the inpatient treatment programme, there was a significant change in mean body mass index (BMI) with a BMI at the start of the group of 14.82 (SD = 1.78) and a mean BMI of 15.56 (SD = 1.84) at the end. All participants were females diagnosed with AN, 66% having restrictive subtype, 20% having binge–purge subtype, and 14% having atypical AN.

**Figure 2:**
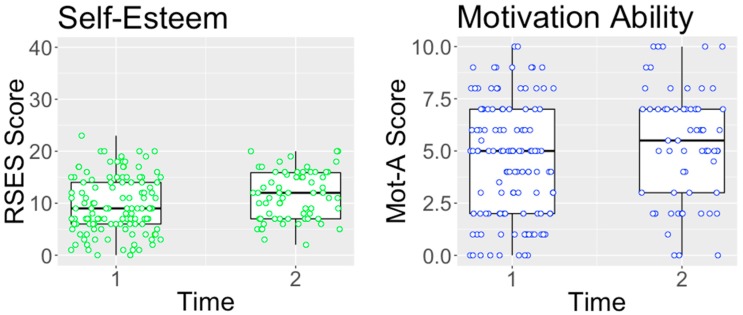
Self-report measures results before and after self-esteem group. RSES, Rosenberg Self-Esteem Scale; Mot-A, Motivational Ruler Ability to Change; Time 1, before self-esteem group; and Time 2, after self-esteem group.

Participants’ scores on the RSES and MR are presented in Figure 2. The findings suggest that patients’ self-reported self-esteem scores signitificantly changed over the duration of the group (RSES) (F(79) = 21.5, *p* < 0.001, ES = 0.39) with patients scoring on average 9.55 (SD = 5.42) at the start of the intervention and then 11.57 (SD = 4.62) at the end of the intervention. The change in BMI did not have a significant effect on participants’ performance on the RSES across the time points (F(170) = 1.2, *p* = 0.28), suggesting that changes were not explained by weight gain alone. The intervention did not have a significant impact on self-reported importance to change (F(78) = 0.12, *p* > 0.05), Comparing the estimated marginal means showed that at the start of treatment, patients on average scored M = 7.59 (SD = 2.47) and at the end of treatment, patients scored M = 7.32 (SD = 2.72). However, the intervention did lead to a significant positive change on self-reported ability to change (F(79) = 8.9, *p* < 0.01, ES = 0.31). Comparing the estimated marginal means showed that at the start of the treatment patients scored M = 4.50 (SD = 2.74) and at the end of treatment patients scored M = 5.34 (SD = 2.68).

### 3.1. Secondary Analysis 

The level of ASD traits did not appear to affect the change in RSES or MR scores over time. Patients with high levels of autistic traits (*n* = 21) saw significant positive changes in their self-esteem scores (F(1,20) = −6.63, *p* < 0.01) and motivational ability scores (F(1,20) = −3.06, *p* < 0.01), which is consistent with the main findings. When age is added as a covariate in the analysis, we see a significant effect of age over time on RSS scores (F(114) = 10.5, *p* < 0.05); furthermore, age and RSS scores at the start of the intervention are positively correlated (*r* = 0.32, *n* = 119), suggesting that older participants tended to have higher self-esteem scores at the start. However, age and RSS scores lose their association at the end of the intervention (*r* = 0.21, *n* = 66). This effect was not seen in motivational ability scores, with age having no effect (F(117) = 0.01, *p* > 0.05). Finally, patients’ duration of illness did not affect the change in RSS scores with it having a non-significant interaction (F(88) = 3.3, *p* > 0.05).

### 3.2. Attrition

Since data was collected in a naturalistic setting using routine clinical audit measures, we calculated attrition as those patients who did not complete time 2 measures, suggesting that they had dropped out of the group or clinical programme. The attrition rate based on this method was 45% with 66 patients completing the group and all clinical measures.

## 4. Discussion

This naturalistic treatment evaluation study aimed to explore the impact of a short self-esteem group in a severe and enduring inpatient AN population. Patients saw a statistically significant increase in self-reported self-esteem, which was not associated with weight gain, during the course of the group. This finding broadly supports the work of other studies in this area. It is consistent with previous findings from smaller uncontrolled studies which found that group interventions targeting self-esteem led to improvements in self-esteem among patients with eating disorders, although not exclusively AN [27]. It is also in accordance with two studies that examined the self-esteem of inpatients during regular treatment [28,29]. Furthermore, our findings support other studies that have demonstrated the effectiveness of a CBT-based self-esteem and social skills group among 116 adolescents with AN related disorders [32], suggesting that self-esteem groups could be utilised across age groups.

Conversely, Mehl et al. (2013) have reported an increase in BMI and quality of life after Multi-Family Therapy whilst a decrease in self-esteem [34], possibly because an improvement in eating disorder psychopathology may not be considered as positive by patients [35]. Brockmeyer et al. (2012) have demonstrated that low weight may lead to an increase in self-esteem in patients with AN as losing weight may be experienced as a success [35]. However, our study has demonstrated that an improvement in inpatients’ self-esteem can be seen at posttreatment when their BMI also increased. This suggests that additional therapeutic input could be beneficial throughout inpatient treatment. 

The current self-esteem group was designed to foster awareness and reflection on critical and negative thinking with the aim to shift patients’ negative view of themselves. This is in line with a qualitative study that has explored young peoples’ retrospective view of inpatient treatment for AN and has demonstrated that the majority of patients identified low self-esteem as an important treatment target alongside the focus on weight gain [36]. It also supports the work of Vanderlinden and colleagues (2007) who have reported that patients with AN and their carers viewed an increase in self-esteem as a key ingredient in the treatment of AN [37].

The present study did not detect a significant change in patients’ self-reported measure of importance to change. There was, however, a significant change in patients’ self-reported measure of perceived ability to change, with a small effect. This suggests that in relatively chronic patients (mean duration of illness was 9 years) with low BMI (M = 14), shifting self-esteem and confidence to change in brief group formats deserves further attention. 

### Limitations

This study used a naturalistic cohort design in an eating disorder specialist clinical service. The study provides useful insight on a brief group intervention for self-esteem which is in accordance with NICE guidelines (NICE, 2017) suggesting that brief inpatient, evidence-based treatment protocols are welcomed contributions to clinical service provision [38]. However, the absence of a control group from this naturalistic study prevents us from drawing firm conclusions. Furthermore, we were also not able to control for other treatment effects such as refeeding, other therapeutic groups, and individual therapy input. Another limitation of the study is that this particular inpatient ward is female only and therefore, no males participated in this study; future studies should examine the effect of self-esteem groups on males with AN.

## 5. Conclusions

In conclusion, self-esteem improved for patients with AN over the duration of a brief CBT-based self-esteem group. With so few studies focused on self-esteem available in the literature, there is a need for further research, especially those that utilize a controlled design. The self-esteem, amongst other group interventions, can be a helpful element to inpatient treatment programmes where the length of stay is short or unknown and when multi-session individual interventions are limited. It can also be useful when patients are severely unwell and therefore may not be able to engage in deep psychological work.

## Figures and Tables

**Figure 1:**
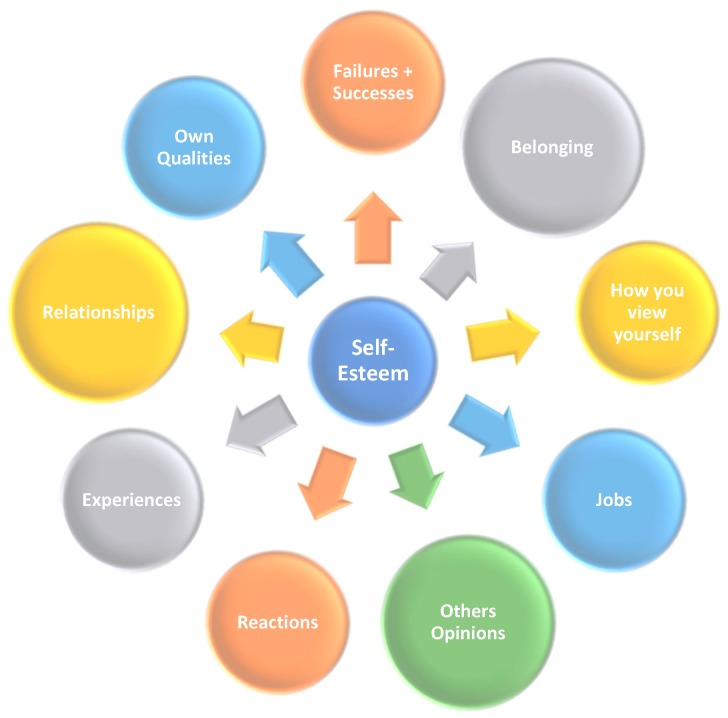
Example of group mind map from session 1.

**Table 1:** Publications evaluating self-esteem groups.

Author/Date	Module	*N* (Number of the Participants)	Outcome Measures	Main Findings
Newns, K.; Bell, L.; Thomas, S. (2003) [27]	Cognitive Behavioural Therapy (CBT)-informed Self-Esteem Group	41	The Eating Attitudes Test-26 (EAT-26)	Significant improvements in self-esteem, depression, and eating attitudes were observed.
			The Beck Depression Inventory (BDI)	
			The General Health Questionnaire (GHQ)	
			The Robson Self-Esteem Self-Report Inventory (RSESRI)	
Karpowicz, E.; Skärsäter, I.; Nevonen, L. (2009) [28]	Inpatient Treatment Programme	38	The Rosenberg Self-Esteem Scale (RSES)	Self-esteem, BMI, weight phobia, and body dissatisfaction improved significantly between pre- and posttreatment.
			Three Subscales (weight phobia, body dissatisfaction, and ineffectiveness) of Eating Disorder Inventory-2 (EDI-2)	
			Body mass index (BMI)	
Lázaro, L.; Font, E.; Moreno, E.; Calvo, R.; Vila, M.; Andrés-Perpiñá, S.; Canalda, G.; Martínez, E.; Castro-Fornieles, J. (2010) [30]	CBT-based Self Esteem Group Therapy and Social Skills Group Therapy	116	The Piers–Harris Children’s Self-Concept Scale (PHC-SCS),	Significant improvements in perceptions of physical appearance, self-concept related to weight and shape and to others, happiness and satisfaction, social withdrawal, and leadership were observed.
			The Self-Esteem in Eating Disorders Questionnaire (SEED)	
			The Socialization Battery (BAS-3)	
Fleming, C.; Doris, E.; Tchanturia, K (2014) [12]	CBT-based Self-Esteem Group	63	The Rosenberg Self-Esteem Scale (RSES)	A significant difference (a decrease) in the perceived importance to change between pre- and post-intervention were observed.
			The motivational ruler	
Collin, P.; Karatzias, T.; Power, K.; Howard, R.; Grierson, D.; Yellowlees, A. (2016) [29]	A specialist inpatient treatment programme	80	The Eating Disorder Examination (EDE)	Statistically significant improvements were found for BMI and all EDE subscales at posttreatment. Statistically significant improvements were also detected for MSEI global and all subscales of MSEI.
			The Multidimensional Self-Esteem Inventory (MSEI)	

**Table 2:** Summary of the structure for group sessions 1–5 [12].

Self-Esteem Group		
Session Number	Session Outline	Homework Tasks
1	Introduction and ground rules	Self-awareness exercise to notice triggers for feeling worse about yourself or Antecedent-Behaviour -Consequence (ABC ) diary to identify thinking patterns and behaviours
	Group mind map: What is self-esteem? (Figure 1)	
	Psychoeducation: Branden’s definition of self-esteem (confidence and worth)	
	Group discussion: What impacts on self-esteem?	
	Complete measures	
	Psychoeducation: Link between self-esteem and eating disorders, short and long term consequences of Eating Disorder on self-esteem	
2	Check-in and homework review	Notice the poisoned parrot and question it—is it fact or opinion? and/or try covering the cage visualisation
	Psychoeducation: Blocks to self-confidence; Introducing the poisoned parrot metaphor for self-critical thoughts	
	Mind map—impact of listening to the poisoned parrot?	
	Cognitive antidote 1: Cover the cage (visualisation)	
	Cognitive antidote 2: Questioning the bird brained thinking (thought challenging)	
	Psychoeducation: Fact vs. opinion	
3	Check-in and homework review	Have a go at neutralising the poisoned parrot using the worksheet or mood tools phone application.
	Psychoeducation: Introduce categories of unhelpful thinking habits	
	Group task: Using scenarios to identify types of unhelpful thinking habits and encourage reflection on individual’s own thinking habits	
	Cognitive strategy: Introduce “neutralising the parrot” thought challenging worksheet and work through example scenarios	
4	Check-in and homework review	Try self-esteem affirmations and/or Achievement, Closeness, Enjoyment (ACE) log—do you spend time doing things which make you feel good?
	Building the alternative: Introduce proud peacock metaphor to balance poisoned parrot	
	Experiential activity: Guided relaxation incorporating covering the cage visualisation and positive affirmations followed by group discussion: Debrief and reflections	
	Cognitive strategy: Introduce positive qualities log, individuals attempt to complete own log	
	Behavioural strategy: Discuss impact of behaviour on self-esteem; introduce pleasurable activities and Achievement, Closeness, Enjoyment (ACE) log	
5	Check-in and homework review	Take home messages—what will you implement in daily life from this group?
	Assertiveness strategy: Our rights questionnaire	
	Groups discussion—do you live by these rights?	
	Psychoeducation: How lack of assertiveness maintains low self-esteem	
	Psychoeducation: Introduce 3 communication methods (passive, aggressive, assertive) and assertiveness skills	
	Group task: Discuss scenarios or role play—how to apply assertiveness skills	
	Complete verbal/written feedback forms and measures	
	Check out	
